# Dark Matter and Mirror World †

**DOI:** 10.3390/e26040282

**Published:** 2024-03-26

**Authors:** Rabindra N. Mohapatra

**Affiliations:** Maryland Center for Fundamental Physics, Department of Physics, University of Maryland, College Park, MD 20742, USA; rmohapat@umd.edu

**Keywords:** mirror world, asymmetric inflation, matter–dark matter coincidence, helium dominated mirror sector

## Abstract

Overwhelming astronomical evidence for dark matter and absence of any laboratory evidence for it despite many dedicated searches have fueled speculation that dark matter may reside in a parallel universe interacting with the familiar universe only via gravitational interactions as well as possibly via some ultra-weak forces. In this scenario, we postulate that the visible universe co-exists with a mirror world consisting of an identical duplicate of forces and matter of our world, obeying a mirror symmetry. This picture, motivated by particle physics considerations, not only provides a natural candidate for dark matter but also has the potential to explain the matter dark matter coincidence problem, i.e., why the dark matter content of the universe is only a few times the visible matter content. One requirement for mirror models is that the mirror world must be colder than our world to maintain the success of big bang nucleosynthesis. After a review of the basic features of the model, we present several new results: first is that the consistency between the coldness of the mirror world and the explanation of the matter dark matter coincidence implies an upper bound on the inflation reheat temperature of the universe to be around $10^{6.5}$ GeV. We also argue that the coldness implies the mirror world consists mainly of mirror Helium and very little mirror hydrogen, which is the exact opposite of what we see in the visible world.

## 1. Introduction

There is now overwhelming evidence in favor of the existence of dark matter from many astrophysical observations such as the speed of galaxies in the Coma cluster, flat rotation curves of stars in galaxies, as well as the Chandra image of two galaxies crossing each other in the Bullet Cluster with dark matter moving ahead of the visible matter. This conclusion seems to have been further confirmed by the study of the cosmic microwave spectrum obtained by the NASA WMAP spacecraft followed by other space missions such as the Planck spacecraft of the European Space Agency, etc. This has granted urgency to the question of what dark matter is and if it is a collection of particles spread out over the universe, what particles are they and what kind of forces they experience other than gravity. The hope is that any understanding of dark matter will provide a glimpse into the nature of physics beyond the standard model.

Experiments in the laboratory set-ups deep underground as well as in colliders have been ongoing for the last thirty years to obtain the dark matter particle (or particles) with more and more sophisticated techniques, but they have all ended up with negative results (for a review, see [1]; see, however, the claims by the DAMA collaboration [2]). This has fueled speculation that dark matter could be residing in a parallel universe (or the mirror universe), in which case its only interaction with known matter (i.e., protons, neutrons or electrons) is via gravity forces or similar ultra-weak forces. This would explain why it seems to elude discovery by conventional detectors. It is the ramification of this idea that we discuss in this article. We call the familiar proton, neutron and electron the visible particles and their mirror partners the mirror protons ($p^{\prime }$), mirror neutrons, etc.

The idea of a mirror universe first appeared in the famous parity violation paper of Lee and Yang in 1956 [3], where they noted that while parity is violated maximally in the beta decay process in our universe, it could be a good symmetry of nature if there was another sector to our universe (called mirror world here) with identical particle spectra and force content to our sector, with the opposite chirality fermions in the mirror sector participating in the mirror beta decay. Our world and the mirror world would transform to each other under mirror (or $Z_{2}$) symmetry. This picture is also motivated by a class of string theories based on the $E_{8}\times E_{8}^{\prime }$ group.

This picture provides a minimal extension of the standard model with very few additional parameters describing it. The phenomenological implications of this hypothesis were first discussed in a paper by Kovzarev, Okun and Pomeranchuk [4] in 1966. In recent years, these models have been the focus of many papers in the context of both particle physics and cosmology [5,6,7,8,9,10,11,12]. In particular, this model also provides a natural candidate for dark matter, which is the main motivation in this paper. The dark matter particle could either be mirror hydrogen or mirror neutron [13,14,15,16,17], whichever is the lightest baryonic particle. We choose the mirror neutron alternative here.

In this model, the dark matter displays self-interaction, which seems to be a useful attribute to explain several puzzles of the collisionless dark matter hypothesis [18,19].

After summarizing the basic ingredients of this model, the paper focuses on two of its salient features: (i) first is an important consistency requirement, which says that the mirror world must be cooler than ours. We outline how this can possibility be realized in concrete models; (ii) next, we present a scenario which provides a resolution [20] of the coincidence problem of matter and dark matter. For another recent proposal in this direction, see [21]. These scenarios require that the mirror fermions must have a higher mass than the fermions of our world. We then discuss the implications of these two ingredients for structure in the mirror universe.

The main new results of this paper are the following: (i) for the first time, we demonstrate the consistency between the colder mirror world and the scenario for matter–dark matter coincidence. (ii) This consistency requirement implies that the inflation reheat temperature of the universe must be less than $10^{6.5}$ GeV. (iii) A final interesting result is that the combined effect of lower temperature and higher mirror electroweak VEV implies that the mirror world consists mainly of mirror helium and very little mirror hydrogen, which is exactly the opposite of the situation in our universe.

## 2. The Mirror Model

As noted in the previous section, the mirror model consists of two sectors to our universe invariant under a discrete $Z_{2}$ symmetry, the mirror symmetry, which transforms all particles and forces of one sector to those of the other. The symmetry guarantees that the particles and forces in the mirror sector of the universe are duplicates of those in the visible sector with equal coupling strengths for the mirror-duplicated forces prior to symmetry breaking. In Table 1, we display the particle content of the model. The symmetry breaking may introduce differences between the two sectors. Depending on the way the gauge symmetries are broken, one can define two broad classes of mirror models. The first is called the symmetric mirror model, where the weak scale in both sectors are the same, whereas the second realization is one where the weak scales in the two sectors are different. In the symmetric mirror model, the visible particles in our world have the same or nearly the same mass as their mirror partners. This can lead to a new class of phenomena such as neutron–mirror-neutron oscillation [22] if there are interactions connecting *n* to $n^{\prime }$ such as $uddu^{\prime }d^{\prime }d^{\prime }$. There are now several experiments searching for this process [23,24]. The Kaon oscillation can now involve four mesons $\left(K,\bar{K},K^{\prime },\bar{K^{\prime }}\right)$, if there are operators of type $\bar{d}s\bar{d^{\prime }}s^{\prime }$ in the theory. Note that such new oscillations are not generic to mirror models and need additional assumptions.

To implement other details such as $n^{\prime }$ as dark matter, we must make sure that $n^{\prime }$ is the lightest baryon in the mirror sector. A single Higgs doublet in each sector leads to relation $\frac{m_{p}}{m_{n}}=,\frac{m_{p^{\prime }}}{m_{n^{\prime }}}$. To make $m_{n^{\prime }}$ less than $m_{p^{\prime }}$, we need to add another Higgs doublet to each sector. Furthermore, to understand neutrino masses, we add a Y=2 triplet Higgs field to each sector as well [25]. Finally, we add three gauge singlet fermions $N_{a}$ connecting the mirror world and the visible one to explain the matter–dark matter coincidence puzzle.

The interactions of the mirror particles are identical duplicates of those in the visible sector, and we do not write them down here.

## 3. Consistency Requirements for the Mirror World Picture

The basic picture for the mirror world scenario is that at the big bang origin time, both ours and the mirror universe were present and started evolving in a completely identical manner. The next big event in the evolution of the universe was the inflation to explain the isotropy, homogeneity, causal connectedness and flatness of the universe. The question that now arises is the whether both the worlds inflate and reheat the in same way. It turns out that they do, but they must reheat to different temperatures after inflation with the mirror world reheating to a cooler temperature and remaining colder for the rest of its life. Thus, the two requirements for mirror models are: (i) asymmetric inflation [12,13,26] so that the reheat temperature in the mirror sector is lower and (ii) the absence of interactions connecting both worlds that will put them in equilibrium with each other after the reheat.

The reason why the mirror sector has to be colder is the fact that the mirror sector adds three extra neutrinos, the mirror electron and a mirror photon to the cosmic plasma of relativistic particles on top of the already known neutrinos, electron and the photon, thus doubling the relativistic degrees of freedom at the BBN epoch. This increased number is in sharp contradiction to the fact that the known neutrinos, the photon and the electrons are just enough to explain the observed helium, deuterium and lithium abundance in the visible universe. The extra degrees of freedom, if any, are collectively denoted by $\Delta N_{eff}$, which is restricted to be less than 0.3. Since the energy density of relativistic particles acts like $T^{4}$, a cooler mirror sector reduces the extra energy density contributed by the extra mirror particles to the desired level to restore consistency of the nucleosynthesis results. The necessary coldness of the mirror sector can be determined from this. If we denote $x=\frac{T^{\prime }}{T}$, the present limits from BBN are satisfied for x≤0.7, assuming $\Delta N_{eff}\leq 0.3$. In the discussion below, we assume x=0.5 for definiteness. The only way to avoid this requirement is to design the model in such a way that all mirror neutrinos, the mirror photon and the mirror electrons are much heavier so that they would have annihilated or decayed away by the BBN epoch. We do not make this assumption in what follows.

To obtain the cooler mirror sector, we resort to the mechanism of asymmetric inflation outlined in [12]. The $Z_{2}$ invariant Higgs potential for the model with a single Higgs doublet in each sector that implements asymmetric inflation is given by

(1) $$\begin{array}{c} V\left(\eta ,H,H^{\prime }\right)=V\left(H,H^{\prime }\right)+m_{\eta }^{2}\eta^{2}+\lambda_{\eta }\eta^{4}+\mu_{\eta }\eta \left(H^{†}H-H^{\prime †}H^{\prime }\right)+\lambda_{\eta H}\eta^{2}\left(H^{†}H+H^{\prime †}H^{\prime }\right) \end{array}$$

with

(2) $$\begin{array}{c} V\left(H,H^{\prime }\right)\;=\;\mu_{H}^{2}\left(H^{†}H+H^{\prime †}H^{\prime }\right)+\lambda_{H}\left[{\left(H^{†}H\right)}^{2}+{\left(H^{\prime †}H^{\prime }\right)}^{2}\right]+\lambda_{H}^{\prime }\left(H^{†}H\right)\left(H^{\prime †}H^{\prime }\right) \end{array}$$

We note that the potential has no $\eta^{3}$ term since η is a mirror $Z_{2}$ odd field. The η-field is the inflaton field which acquires VEV <η>≠0. This asymmetrises η couplings to *H* and $H^{\prime }$ fields leading to $\Gamma \left(\eta \to HH\right)>\Gamma \left(\eta \to H^{\prime }H^{\prime }\right)$ as we see later. After inflation ends, the inflaton field decays to the two sectors in an asymmetric way, leading to different reheat temperatures in the two sectors. The same asymmetric coupling of the η-field also leads to the electroweak VEVs in the two sectors being different. Thus, η plays a dual role in the model, unifying two different aspects of it.

Another way to restore consistency of mirror models with BBN is to add heavy gauge singlet Majorana neutrinos $N,N^{\prime }$ to the two sectors, respectively, and connect them via a mass term $MNN^{\prime }$ + h.c. This can lead to *N* eigenstates with different masses and different couplings to ℓH and $\ell^{\prime }H^{\prime }$ states and a subsequent release of more relativistic particles from their decay to the visible sector compared to the mirror sector [27]. This, in turn, leads to x<1 and solving the BBN problem. We do not follow this route here.

Thus, in our asymmetric mirror scenario, the two parameters that characterize the mirror sector of the universe are *x*, the ratio of the two temperatures and β, the ratio of mass scales, $\beta \equiv \frac{v_{wk}}{v_{wk}^{\prime }}$. One immediate consequence of β≪1 is that the two strong couplings $\alpha_{s}$ and $\alpha_{s}^{\prime }$, which start out being equal at very high energies due to mirror symmetry, become different at low scales, when we obtain $\alpha_{s}\left(\mu \right)\ll \alpha_{s}^{\prime }\left(\mu \right)$. This results from the fact that the mirror top quark decouples much above the visible top quark from QCD running since $\left(m_{t^{\prime }}/m_{t}\right)=\left(v_{wk}^{\prime }/v_{wk}\right)\gg 1$. This leads to the following constraint on QCD and QCD’ scales: $\Lambda_{QCD}^{\prime }\gg \Lambda_{QCD}$, which makes the mirror sector particles, and in particular baryons, heavier.

## 4. Asymmetric Inflation, Weak Scale Asymmetry and Constraints on Model Parameters

As already noted, the η field in Equation (1), which is odd under mirror symmetry, plays an important role in the model; (i) it is the inflaton field; (ii) its VEV asymmetrises the inflaton coupling to the Higgs and mirror Higgs fields, which leads to asymmetric reheating in the two sectors; and (iii) finally, its VEV also asymmetrises the particle spectrum in both sectors and becomes one of the keys to solving the matter–dark matter coincidence problem. We note, parenthetically, that ours is a model of chaotic inflation, which is slightly outside the Planck CMB data, but this problem can be cured by coupling η non-minimally to gravity. We do not dwell on this aspect here. As an order of magnitude, we estimate that VEV of η to be of order $|M_{\eta }|$, which is assumed in the discussion below.

We see that η vev makes the *H* and $H^{\prime }$ masses different as follows [28]:

(3) $$\begin{array}{c} M_{H}^{2}=\mu_{H}^{2}+\lambda_{\eta H}v_{\eta }^{2}+\mu_{\eta }v_{\eta }\\ M_{H^{\prime }}^{2}=\mu_{H}^{2}+\lambda_{\eta H}v_{\eta }^{2}-\mu_{\eta }v_{\eta } \end{array}$$

The reheat temperatures are given by $T_{RH}≃\sqrt{\Gamma_{\eta }M_{P}}$. If we then use the width of η→HH, etc., as (assuming $M_{\eta }\gg \mu_{H}$)

(4) $$\begin{array}{c} \Gamma \left(\eta \to HH\right)≃\frac{{\left(\mu_{\eta }+\lambda_{\eta H}v_{\eta }\right)}^{2}}{8\pi M_{\eta }} \end{array}$$

and similarly for $\eta \to H^{\prime }H^{\prime }$ decay, we obtain

(5) $$\begin{array}{c} \frac{T_{RH}^{\prime }}{T_{RH}}∼\frac{\mu_{\eta }-\lambda_{\eta H}v_{\eta }}{\mu_{\eta }+\lambda_{\eta H}v_{\eta }}\approx 0.3 \end{array}$$

We now summarize the constraints on the parameters of the potential above that follow from weak scale asymmetry and asymmetric reheating. For the sake of illustration, we take $M_{\eta }$∼$10^{8}$ GeV as a benchmark parameter.

- First, we find that <η>∼$M_{\eta }$ for $\lambda_{\eta }$∼1.
- Since $M_{H,H^{\prime }}^{2}$ to break the electroweak symmetries in both the visible and mirror sectors, to obtain $v_{wk}^{\prime }$∼$10^{3}v_{wk}$, $|\mu_{H}^{2}+\lambda_{\eta H}v_{\eta }^{2}|$∼$\mu_{\eta }v_{\eta }$∼$10^{10}$ GeV^2^, $\mu_{\eta }v_{\eta }>0$ and $\mu_{H}^{2}<0$ is required, as well as $-|\mu_{H}^{2}|+\lambda_{\eta H}v^{2}-\eta \mu_{\eta }v_{\eta }\approx 10^{4}$ GeV^2^. This produces the desired parameter range for our model, i.e., β∼$10^{-3}$. This also implies that
  (6) $$\begin{array}{c} \mu_{\eta }v_{\eta }\approx \left|\mu_{H}^{2}\right|∼10^{10}{\mathrm{GeV}}^{2} \end{array}$$
- This offers $\mu_{\eta }$∼100 GeV and $\lambda_{\eta }$∼$10^{-6}$ for our benchmark choice.
- We require $\frac{T_{RH}^{\prime }}{T_{RH}}$∼*x*∼0.5. This leads to $\mu_{\eta }\approx 2\lambda_{\eta H}v_{\eta }$.
- These results for $M_{\eta }$∼$10^{8}$ GeV produce $T_{RH}$∼$10^{6.5}$ GeV. Thus, we obtain an upper bound on the inflation reheat in the visible sector.
- The last constraint at this stage is that the mass of the η-field must be such that the $HH\to H^{\prime }H^{\prime }$ scattering via η exchange does not thermalize the two sectors till the BBN epoch, i.e., $T_{BBN}$∼$10^{-3}$ GeV. The condition for this is that $\frac{\mu_{\eta }^{4}T}{8\pi M_{\eta }^{4}}\leq 10\frac{T^{2}}{M_{P}}$. For $M_{\eta }$∼$10^{8}$ GeV, this implies that the temperature below which there is equilibrium is given by $T_{*}$∼$\frac{\mu_{\eta }^{4}M_{P}}{100M_{\eta }^{4}}$∼10 eV. This is acceptable since it does not affect BBN.
- In fact, by varying the value of $M_{\eta }$, we find that this condition implies an upper limit on $T_{RH}$ of order $10^{6.5}$ GeV. In Table 2, we present the value of $T_{*}$ for different choices of $M_{\eta }$ which helps us to obtain this upper limit on $T_{RH}$. This upper limit is important since it implies that the masses of the singlet fermions *N* must be less than this if they have to be present in the universe to generate lepton asymmetry in both the visible and dark sectors (see the next section).

## 5. Matter–Dark Matter Coincidence

As already noted, the lightest baryon of the mirror sector (in our case, $n^{\prime }$) can be a dark matter of the universe and in the framework described below is an asymmetric dark matter [29,30]. After the end of inflation reheat, the universe undergoes usual Hubble expansion and processes leading to leptogenesis and Big Bang Nucleosynthesis start in both sectors. Due to asymmetric weak scales and colder mirror sector, the value of $g^{*}$, the number of degrees of freedom are not always same in both sectors, but for simple illustration of the phenomena we are interested in, we assume them to be same.

Let us first discuss how the mirror world explains the matter–dark matter coincidence puzzle via leptogenesis [12]. For this purpose, we add three SM singlet Majorana fermions $N_{a}$ portals which connect both sectors of the universe via the following couplings [12]:

(7) $$\begin{array}{c} L_{Y}\;=\;M_{N}\left(NN\right)+hN\left(LH+L^{\prime }H^{\prime }\right)+h.c. \end{array}$$

where we drop the three flavor indices in coupling matrix *h* and mass matrix $M_{N}$. We then use leptogenesis as the co-genesis mechanism for matter and dark matter following [12] (we note that there is no ηNN coupling in the theory, since NN is $Z_{2}$ even when η is $Z_{2}$ odd). We assume that the mass of the *N* singlets is $10^{6}$ GeV so that after reheating is completed they exist in the cosmic fluid. They connect with the SM and mirror particles via their couplings in Equation (5). We further assume that they produce lepton asymmetry via leptogenesis in both the visible and mirror sectors. Due to mirror symmetry, they produce an equal amount of lepton asymmetry in both sectors, which is then converted to both visible and mirror baryons, producing $n_{B}=n_{B^{\prime }}$ due to their respective sphaleron interactions. The lepton asymmetry is produced below *T*∼$M_{N}$∼$10^{6}$ GeV, when despite a colder mirror sector, the mirror sphalerons are still active. This requirement also puts an upper limit on $v_{wk}^{\prime }\leq M_{N}$.

Since the *N* masses are low, the mechanism is the resonant leptogenesis mechanism [31], which requires that at least two of the portal right-handed neutrinos (RHN) are degenerate. The RHNs must exit the equilibrium at $T≃M_{N}$. The condition for that is

(8) $$\begin{array}{c} \frac{hh^{†}M_{N}}{4\pi }≃10\frac{M_{N}^{2}}{M_{P}} \end{array}$$

For $M_{N}$∼$10^{6}$ GeV, this implies *h*∼$10^{-5}$. In resonant leptogenesis, by adjusting the degree of degeneracy, one can produce an adequate amount of lepton asymmetry.

The addition of the cogenesis to the mirror model imposes these constraints on the model parameters:

- We must guarantee that the wash-out processes are out of equilibrium, which requires that $K=\frac{\Gamma }{H}\leq 10^{6}$, which is easily satisfied in the model.
- We must also ensure that the *N*-mediated $\ell H\to \ell^{\prime }H^{\prime }$ scatterings do not equilibrate the two worlds. This implies that
  (9) $$\begin{array}{c} \frac{{\left(hh^{†}\right)}^{2}T^{3}}{4\pi M_{N}^{2}}<\frac{10T^{2}}{M_{P}} \end{array}$$
  This condition is easily satisfied below the reheat temperature of $10^{6.5}$ GeV.

It follows from these considerations that $n_{B}=n_{B^{\prime }}$. Therefore, if the mass of the mirror neutron dark matter is about 4–5 GeV, we have an understanding of the matter–dark matter coincidence puzzle.

## 6. Helium Universe in the Mirror Sector

In this section, we discuss some implications of the two outstanding features of the asymmetric mirror model, i.e., ($\beta \equiv \frac{v_{wk}}{v_{wk}^{\prime }}<1$ ) and a cold mirror sector ($x\equiv \frac{T^{\prime }}{T}<1$). As a first step, we discuss the consequences of nucleosynthesis in the mirror sector. We present a very simple approximate analysis, neglecting the difference in the degrees of freedom between the two sectors as the universe evolves and also subtleties associated with deuterium formation prior to helium synthesis.

First, we need to write down the expansion rate equation of the universe in terms of the temperature of the mirror sector, $T^{\prime }=xT$, where *T* is the temperature of the visible sector. We then have

(10) $$\begin{array}{c} M_{P}^{2}H^{2}=g^{*}{T^{\prime }}^{4}\left(1+x^{-4}\right) \end{array}$$

To discuss big bang nucleosynthesis (BBN) in the mirror sector [32], we first find out the value of $T^{\prime }$ at which the mirror weak reactions involving mirror neutrinos, such as $\nu^{\prime }+n^{\prime }\to p^{\prime }+e^{\prime }$, that maintain the mirror neutron proton equality go out of equilibrium. The equation for that is

(11) $$\begin{array}{c} {G_{F}}^{2}\beta^{4}{T^{\prime }}^{5}\leq \frac{{g^{*}}^{1/2}{T^{\prime }}^{2}{\left(1+x^{-4}\right)}^{1/2}}{M_{P}} \end{array}$$

As just noted, we assume the number of degrees of freedom $g^{*}$ to be same in each sector for simplicity. Adding extra details on this would not significantly change our broad conclusion.

This leads to the $\nu^{\prime }$ decoupling temperature $T_{*}^{\prime }$ in the mirror sector to be

(12) $$\begin{array}{c} T_{*}^{\prime }≃{\left(g^{*}\right)}^{1/6}G_{F}^{-2/3}\beta^{-4/3}{\left(1+x^{-4}\right)}^{1/6}{M_{P}}^{-1/3}\;\mathrm{GeV} \end{array}$$

For our parameter choice, this $n^{\prime }/p^{\prime }$ freeze-out occurs at the mirror sector temperature equal to ∼70 GeV. It was shown in Ref. [20] that for a certain choice of the two Higgs doublet VEVs (or tan $\beta^{\prime }$) in the model, the mirror neutron can be lighter than the mirror proton and the mass difference $m_{p^{\prime }}-m_{n^{\prime }}≃2$ GeV or so, which means that the number of mirror protons and mirror neutrons at their freeze-out epoch is about same, with the number of neutrons slightly exceeding that of protons. Since the nuclear forces in the visible and the mirror sectors are similar, we expect that all the protons ($p^{\prime }$) combine with equal number of neutrons ($n^{\prime }$) to form mirror Helium with very few mirror neutrons left over leading to a Helium dominated mirror universe as announced. Dark matter then consists of mirror helium and leftover mirror neutrons (for an interpretation of the DAMA results in the mirror model framework, see [33,34]).

## 7. Comments and Conclusions

In this brief note, we summarize the main points of the asymmetric mirror world model for dark matter, where the electroweak symmetry breaking in the mirror sector is higher than that of the visible sector. It turns out that the consistency between a colder mirror sector with electroweak VEV asymmetry implies an upper bound on the inflation reheat temperature of $10^{6.5}$ GeV, which is a new result of this paper. We outline the co-genesis for matter and dark matter in this set-up and show that the weak scale asymmetry, together with a colder mirror sector, leads to the mirror sector being helium-dominated. This has implications for structure formation in the mirror universe.

There are many relevant points about asymmetric mirror models that we do not discuss here. For example, in these models, there are other gauge-invariant interactions which can connect both sectors, e.g., photon mirror photon mixing coming from hypercharge gauge boson mixings $B_{\mu \nu }B^{\prime \mu \nu }$, Higgs mixings $H^{†}H{H^{\prime }}^{†}H^{\prime }$, etc. For the consistency of the model described here, these interactions must be highly suppressed. The other issue that we do not address is the formation of structure in a helium universe and mirror stellar evolution as well as the possibility that familiar neutron stars could contain mirror dark matter in their core and how it can affect their evolution, the latter item discussed in [35,36,37].

## Figures and Tables

**Table 1:** Gauge quantum numbers of all the fields in the theory; η is a mirror parity odd field.

Our World	$\mathrm{SU}{\left(3\right)}_{c}\times \mathrm{SU}{\left(2\right)}_{L}\times U{\left(1\right)}_{Y}$	Mirror World	$\mathrm{SU}{\left(3\right)}_{c}^{\prime }\times \mathrm{SU}{\left(2\right)}_{L}^{\prime }\times U{\left(1\right)}_{Y}^{\prime }$
Visible fermions		mirror fermions	
$Q_{L}$	(3,2,1/3)	$Q_{L}^{\prime }$	(3,2,1/3)
$u_{R}$	(3,1,4/3)	$u_{R}^{\prime }$	(3,1,4/3)
$d_{R}$	(3,1,−2/3)	$d_{R}^{\prime }$	(3,1,−2/3)
$\ell_{L}$	(1,2,−1)	$\ell_{L}^{\prime }$	(1,2,−1)
$e_{R}$	(1,1,−2)	$e_{R}^{\prime }$	(1,1,−2)
Gauge bosons		Mirror Gauge bosons	
W,Z,γ,Gluons		$W^{\prime },Z^{\prime },\gamma^{\prime },\mathrm{mirror}\;\mathrm{Gluons}$	
Scalar sector		mirror scalar	
*H*	(1,2,1)	$H^{\prime }$	(1,2,+1)
η	(1,1,0)	η	(1,1,0)

**Table 2:** Lowering $M_{\eta }$ brings the mirror and visible sectors to equilibrium and makes the theory unacceptable. Increasing $M_{\eta }$ keeps the theory acceptable but yields a lower $T_{RH}$. Thus, near about the value of $M_{\eta }$∼$10^{8}$, the maximum $T_{RH}$ is yielded. We choose this optimal value for our parameters.

$M_{\eta }$	$\mu_{\eta }$	$T_{\mathrm{RH}}$	$T_{*}$	Comment
$10^{8}$ GeV	100 GeV	$10^{6.5}$ GeV	10 eV	acceptable
$10^{10}$ GeV	1 GeV	$10^{3}$ GeV	$10^{-25}$ GeV	acceptable
$10^{6}$ GeV	$10^{4}$ GeV	$10^{8.5}$ GeV	$10^{9}$ GeV	unacceptable

## Data Availability

No new data were created or analyzed in this study. Data sharing is not applicable to this article.
